# Atypical Presentation of Breast Cancer Metastasis to the Bowel: A Case Report

**DOI:** 10.7759/cureus.53896

**Published:** 2024-02-09

**Authors:** Ayodeji Ayeni, Peter Elemile, John Nwadiokwu, Victor Okebalama, Opeyemi Taiwo

**Affiliations:** 1 Department of Surgery, Montefiore Medical Center, Bronx, USA; 2 Department of Public Health, Olabisi Onabanjo University, Ago-Iwoye, NGA; 3 Department of Surgery, Babcock University Teaching Hospital, Ilishan-Remo, NGA; 4 Department of Histopathology, Babcock University Teaching Hospital, Ilishan-Remo, NGA; 5 Department of Community Medicine and Primary Care, Olabisi Onabanjo University, Ago-Iwoye, NGA

**Keywords:** immunohistochemistry, gastrointestinal symptoms, small and large bowel, metastasis, invasive lobular carcinoma

## Abstract

Breast cancer rarely metastasizes to the intestinal tract. It is even more uncommon to find intestinal metastasis as the first sign of distant spread. We describe an atypical case of small and large bowel carcinomas arising from primary breast cancer and presenting as the first evidence of distant metastasis. Clinicians should therefore consider the possibility of gastrointestinal (GI) metastasis when patients with breast cancer present with GI symptoms.

## Introduction

Distant metastasis in breast cancer commonly occurs in the liver, brain, lungs, and bone and is the most common cause of death in individuals with this malignancy. Metastasis in the gastrointestinal (GI) system is an uncommon occurrence [1,2]. While colonic metastases are extremely uncommon, the stomach and small intestine are the most common sites of GI tract involvement [1]. Large intestine metastases may cause perplexing diagnostic issues since they can resemble primary large bowel cancer.

We hereby report a unique case of primary breast cancer spread into the small and large bowel, presenting as the initial sign of distant metastases.

## Case presentation

A 69-year-old woman, diagnosed with left invasive lobular breast cancer (T3N1M0), triple-positive for estrogen receptor (ER), progesterone receptor (PR), and Her2/neu, had a mastectomy, radiotherapy, and adjuvant chemotherapy in 2021. In the follow-up period, she showed no signs of local recurrence. Two years later, she presented with complaints of constipation and vomiting. A physical examination revealed a full, soft abdomen with a supraumbilical nodule and stable vital signs. Barium meal and follow-through showed areas of luminal narrowing in the duodenum, jejunum, ileum, and ileocecal regions with pre-stenotic dilations. She was managed non-operatively for possible radiation enteritis; however, her symptoms persisted. An exploratory laparotomy revealed multiple discrete intraluminal tumors in the jejunum, ileum, caecum, transverse colon and splenic flexure, and peritoneal nodules in the mesentery of the small and large intestines for which she had jejunoileal resection with end-to-end jejunoileal and side-to-side ileosigmoid anastomoses.

**Figure 1:**
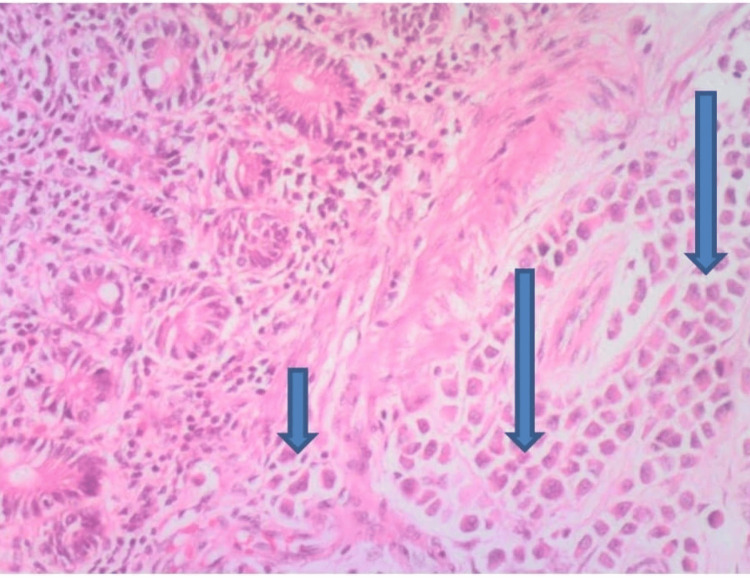
H and E at x100 showing clusters of malignant epithelial cells infiltrating the GIT submucosal layer (long arrow) and muscularis mucosae (short arrow) suggestive of metastatic carcinoma

**Figure 2:**
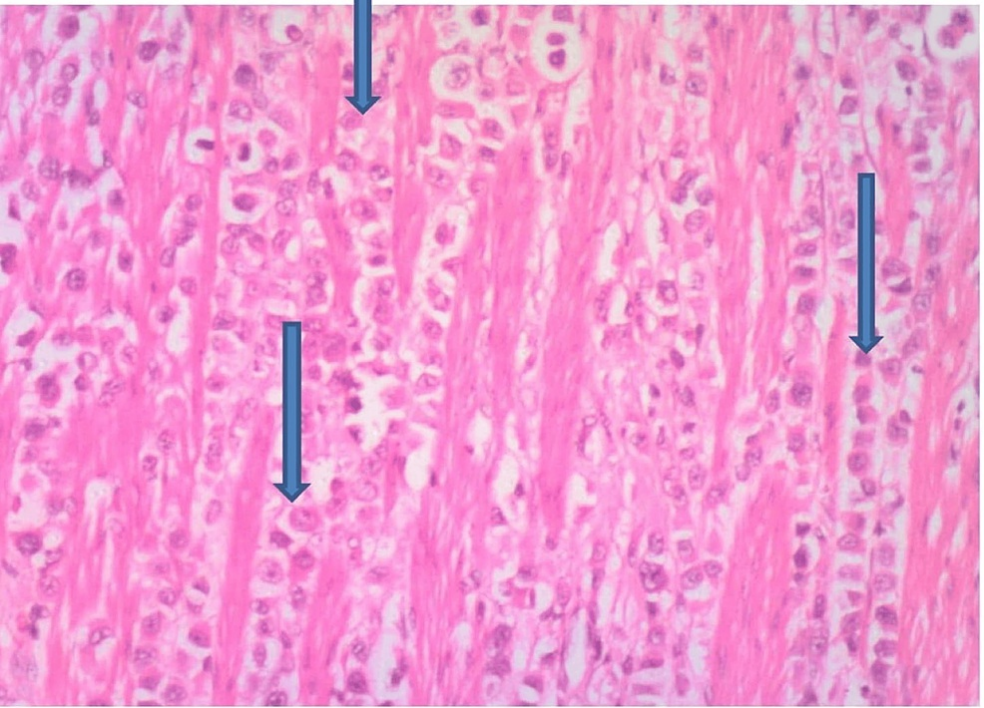
H and E at x100 showing trabeculae of malignant epithelial cells infiltrating and splitting the GIT muscular layer (arrows) suggestive of metastatic carcinoma

**Figure 3:**
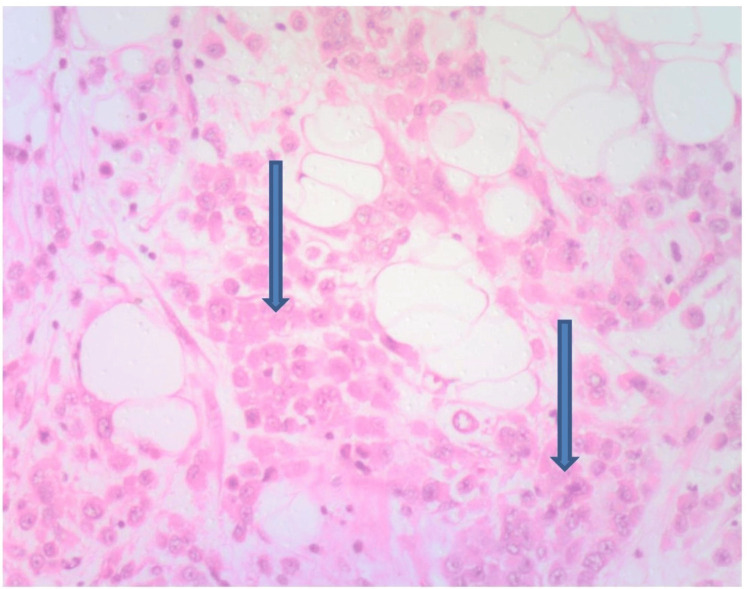
H and E at x100 showing nests of malignant epithelial cells infiltrating the GIT serosal layer (arrows) suggestive of metastatic carcinoma

Histology of the jejunoileal segment revealed an infiltrating malignant lesion with tumor cells arranged in sheets, nests, and trabeculae consistent with metastatic adenocarcinoma, most likely from breast primary (Figures 1-3).

**Figure 4:**
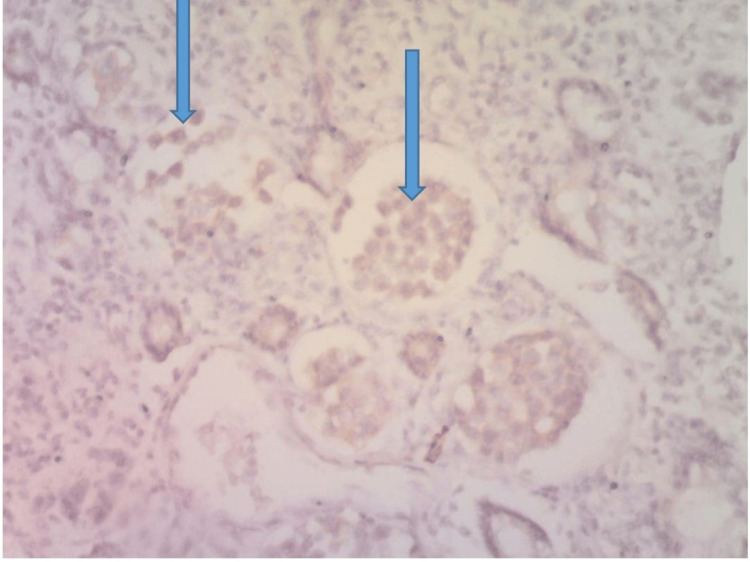
HER 2 immunohistochemical stain @x100 showing mild cytoplasmic staining of the tumor cells (arrows) consistent with metastatic carcinoma from breast primary

**Figure 5:**
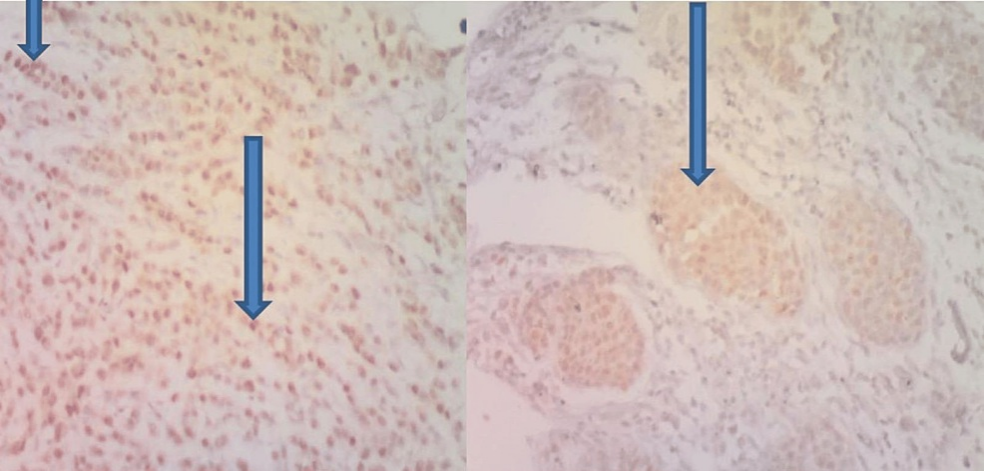
ER immunohistochemical stain @ x100 showing moderate-to-strong nuclear staining of the tumor cells (arrows) consistent with metastatic carcinoma from breast primary

Immunohistochemistry for ER showed about 80% of the tumor cells having moderate to strong nuclear positivity, while about 30% showed mild cytoplasmic positivity with Her 2 stain (Figures 4-5). The diagnosis of metastatic carcinoma to the GI tract from an invasive primary carcinoma of the breast (luminal B molecular subtype) was made.

On the 14th postoperative day, she developed recurrent bowel obstruction and was managed conservatively. Days later, she was found to be tachypneic with chest x-ray features of pulmonary metastasis and, unfortunately, died on the 28th postoperative day.

## Discussion

Breast cancer is the most common cause of cancer-related death among women globally, with mortality being disproportionately higher in Africa with a mortality-to-incidence ratio of 0.44 [3]. Metastasis is a leading cause of mortality and occurs commonly in the bones, lungs, liver, and brain. Intestinal metastasis is rare. Bolzacchini et al. reported 96 cases over a 45-year period [2]. Additionally, among reported cases, it is not uncommon to find these intestinal deposits in patients with prior documented spread to other organs or background carcinomatosis [1,4,5]. However, in our patient, metastasis was first detected in the bowel, which added to the diagnostic dilemma.

The reported median interval between breast cancer diagnosis and presentation with intestinal metastasis is variable. D'Angelo et al. and McLemore et al. reported five and seven years, respectively, in different studies [6,7]. Our patient, in contrast, presented two years after diagnosis, although she had a late-stage disease at diagnosis. Few individuals survive for more than 24 months following a diagnosis of GI metastases, indicating low survival rates [1]. This was the unfortunate case of our patient.

Most studies have identified lobular carcinoma as the most common histological type of breast cancer associated with intestinal metastasis [1,2,4,7]. In a survey by Borst et al., 4.5% of metastasis from lobular carcinomas was to the GI tract, compared to 0.2% for ductal carcinomas [8]. This finding is consistent with the histological type seen in our patient.

Primary intestinal tumors should be differentiated from metastatic bowel disease. Histopathological and immunohistochemical similarities to a primary disease outside the GI tract are in keeping with metastatic disease. Staining for markers such as GCDFP-15, ER, PR, HER-2-neu, GATA-3, CK5/6, and CK7 can help determine breast origin with variable levels of sensitivity and specificity [7,9,10]. Additionally, multiple synchronous primary intestinal tumors involving the small and large bowel are infrequent, making diagnosing metastatic disease more plausible in patients presenting with a known breast cancer history. Nevertheless, bowel metastasis from primary mammary carcinoma may present as a solitary mass.

Clinical presentation is variable. Some patients present with signs of bowel obstruction, bleeding, perforation, or symptoms similar to other GI conditions such as radiation enteritis and inflammatory bowel disease. Radiological findings are often non-specific, and many patients are diagnosed at surgery, during which a palliative surgical intervention may be offered with little or no benefit. There have been some reported benefits with chemotherapy and/or hormonal therapy [1,5]. However, our patient was unfit to receive either.

## Conclusions

Intestinal metastasis from breast cancer is uncommon and may rarely be the first evidence of distant metastasis. This can easily constitute a diagnostic dilemma and, thus, can be easily misdiagnosed. It also has a low survival rate. Therefore, physicians should be alert to the possibility of bowel metastasis in patients with a medical history of breast cancer, especially of the lobular type, presenting with gastrointestinal symptoms, even as this may improve clinical outcomes.
